# Determination of Cloud-top Height through Three-dimensional Cloud Reconstruction using DIWATA-1 Data

**DOI:** 10.1038/s41598-020-64274-z

**Published:** 2020-05-05

**Authors:** Ellison Castro, Tetsuro Ishida, Yukihiro Takahashi, Hisayuki Kubota, Gay Jane Perez, Joel S. Marciano

**Affiliations:** 1 Faculty of Science, Hokkaido University, Sapporo, Japan; 2 Institute of Environmental Science and Meteorology, University of the Philippines Diliman, Quezon City, Philippines; 3 Advanced Science and Technology Institute, Department of Science and Technology, Quezon City, Philippines; 4 Electrical and Electronics Engineering Institute, University of the Philippines Diliman, Quezon City, Philippines

**Keywords:** Imaging and sensing, Atmospheric dynamics

## Abstract

Cloud-top height is a useful parameter with which to elucidate cloud vertical growth, which often indicates severe weather such as torrential rainfall and thunderstorms; it is widely used in meteorological research. However, general cloud-top height estimation methods are hindered by observational and analytical constraints. This study used data from DIWATA-1, the Philippines’ first microsatellite, to overcome these limitations and successfully produce sophisticated three-dimensional cloud models via stereo-photogrammetry. High-temporal snapshot 200-ms-interval imaging of clouds over Iloilo, Philippines, is performed. Two types of telescopes were used to capture 30 stereoscopic cloud images at ~60- and ~3-m ground sampling resolutions; these were used to construct three-dimensional cloud models with 40- and 2-m vertical resolutions, respectively. The imaged clouds have heights of 2.0 to 4.8 km, which is below freezing level for the Philippines and typical of stratocumulus and cumulus clouds. The results are validated using cloud-edge heights determined by measuring the distance from the clouds to their ground shadows. An RMSE of 0.32 km and a maximum difference of 0.03 km are found for the low- and high-resolution telescopes, respectively. For further validation, the results are compared with cloud-top heights estimated from HIMAWARI-8 images captured on the same day, yielding an average vertical difference of 0.15 km and a maximum difference of 1.7 km.

## Introduction

The vertical growth of clouds has been employed as an indicator of severe weather conditions such as torrential rainfall and thunderstorms in many vital meteorological studies. One important parameter related to vertical cloud growth is cloud-top height. Specifically, higher cloud-top heights are directly related to heavier rainfall^1,2^ and have also been linked to temperature and moisture layer altitudes, which are closely related to tropical cyclone intensity^3–6^. It is also known that cloud-top height is a key parameter for determining thunderstorm intensity through observations of growth rate^7^. Over the past few decades, several observational and analytical techniques, such as the Light Detection and Ranging (LIDAR), atmospheric absorption and bispectral methods, the use of cloud shadow, and stereoscopic measurements^8^, have been utilized to estimate cloud-top heights.

In this study, DIWATA-1—the Philippines’ first microsatellite—uses a stereoscopic technique to measure cloud-top heights. Taking stereoscopic measurements from space is not a new technique; it has been employed by geostationary satellites, which are located at two different but nearby locations, such as the Geostationary Operational Environmental Satellite system (GOES)^7^, METEOSAT^9^, and the Geostationary Meteorological Satellite (GMS)^10^. It has also been used by single satellites carrying multiple cameras that have different viewing angles, such as the Multiangle Imaging Spectroradiometer (MISR)^11^, Advanced Spaceborne Thermal Emission and Reflection Radiometer (ASTER)^12^, and the Along Track Scanning Radiometer (ATSR)^13,14^. The primary advantage of DIWATA-1, among these satellites and sensors, is its high spatial resolution which can be 58.1 m and/or 2.9 m, and its capability to capture stereoscopic images with a high temporal resolution of 200 ms. The other satellites and sensors have spatial resolutions of only 1 km^7,9,11,13^, with the exception of ASTER, which has a resolution of 15 or 90 m^12,15^, and reported time intervals of 2.5 to 7.5 min^7,9,11,16,17^. Furthermore, vertical accuracy reported from past studies are typically 0.5 km^7,9–12^ to 1 km^13,14^. With the higher spatial resolution of DIWATA-1, the vertical resolution of a cloud-top height measurement also increases due to the decrease in the error of the disparity between stereoscopic images^18^. The shorter time interval between the image pairs further decreases the error due to cloud advection and changing morphology.

Other cloud-top height measurement techniques such as using the cloud brightness temperature (or bispectral method)—where the height (pressure) is determined by comparing the brightness temperature with vertical sounding from a radiosonde or sounder^19,20^—have relatively low spatial resolution imagery^15,21,22^. However, the vertical accuracy of this method can also reach 1.1 km^20,23,24^, depending on the emissivity of the clouds and accuracy of the vertical profile^8,25^. Using the atmospheric absorption bands of CO_2_ and O_2_ also results in a relatively low spatial resolution^25,26^ and a reported vertical accuracy of 0.1 km to 1.5 km^25,27,28^. Furthermore, this technique is based on the absorption difference, which varies slightly, of the cloud at two or more wavelengths^29^. LIDAR and radar measurements are the sensors with the highest vertical resolution, as these can range from 7.5 m to 750 m^30,31^. However, these active sensors are technologically complex and expensive^32,33^.

The main goal of this study is to propose a methodology for and assess the capability and consistency of the DIWATA-1 microsatellite to measure cloud-top heights by constructing three-dimensional models of clouds through stereoscopy. The measured cloud-top heights are then compared with HIMAWARI-8’s 11.2 μm (Band-14) and with cloud-edge heights obtained by measuring the distances from the clouds to their corresponding shadows.

## Instrument and Observations

### Instrument

DIWATA-1 is a 50-kg microsatellite built by Filipino students in the Philippine Microsat (PHL-MICROSAT) Program in collaboration with Hokkaido University and Tohoku University. It was launched into an orbital altitude of 403 km from the Kibo module of the International Space Station (ISS) on April 27, 2016^34^. DIWATA-1 carries four optical payloads: a high-precision telescope (HPT) and wide-field camera, both of which are similar to that aboard RISING-2^35^; a space-borne multispectral imager (SMI); and a middle-field camera (MFC). All images have a dimension of 659 by 494 pixels. However, unlike the HPT on RISING-2^35^, the HPT of DIWATA-1 does not include a liquid crystal tunable filter (LCTF); this is integrated in the SMI. In this study, the SMI and the HPT were selected from among the four DIWATA-1 payloads for their advantageous spatial resolutions. The SMI has a pixel resolution of 58.1 m and is a multi-spectral sensor with two charge-coupled devices (CCDs) and captures a spectral image selectively in the visible (400–780 nm) and near-infrared (730–1020 nm) regions at an interval of 1-nm. On the other hand, the HPT has a pixel resolution of 2.9 m and four CCDs that capture images in the blue, green, red, and near-infrared regions. Table 1 shows the specifications of SMI and HPT. With the exposure times given for the SMI and HPT, the actual sampling of the SMI increases from 58.1 m to 94 m while that of the HPT increases to 16 m. One pixel of the SMI sensor can be contaminated by a neighboring pixel, while an HPT pixel can be contaminated by as many as five pixels; this causes a blur in the images.

**Table 1:** Summary of the Specifications of SMI and HPT. The ground sampling distance and field-of-view were estimated at nadir.

	HPT	SMI
Focal Length (mm)	974	48.2
Aperture (mm)	96	17.6
Exposure Time (ms)	2	12
Ground Sampling Distance, GSD (m) *	2.9	58.1
Field-of-View *	1.9 km × 1.4 km (0.29° × 0.22°)	38.3 km × 28.7 km (5.79° × 4.34°)

* The value is estimated based on the altitudes as of image acquisition which is approximately 378.6 km at nadir and at a stationary state.

To avoid the saturation of measured radiances that occur in the visible band of SMI, the 870-nm band with a full width at half maximum (FWHM) bandwidth of 10-nm was used. The HPT images were from the green band, which is centered at 560 nm with ~45-nm FWHM bandwidth. Although two different bands were used in this study, the reflectance of an optically thick or opaque cloud does not vary largely within the scopes of these bands^36–38^.

**Figure 1:**
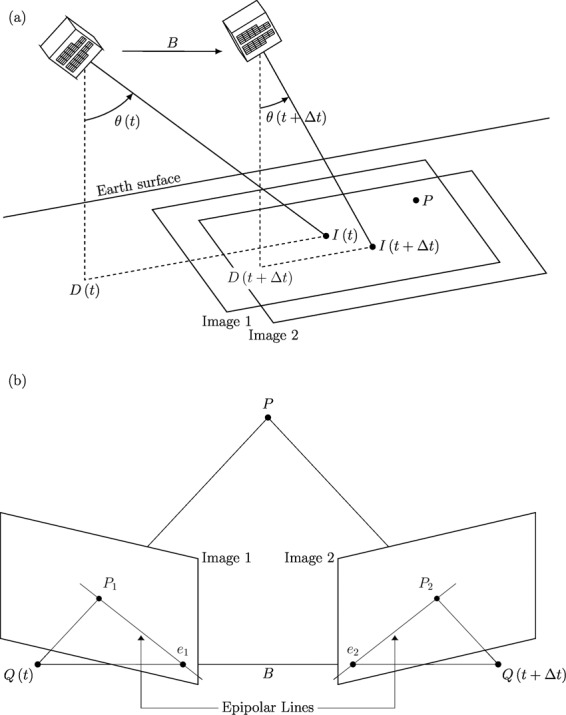
(**a**) DIWATA-1, at 378.6-km altitude as on the day of capture, captured 30 images using both SMI and HPT with a 200-ms time interval between captures. The angles $\theta (t)$ and $\theta (t+\Delta t)$ are those subtended by the subsatellite point and the image centers at times $t\,$and $t+\Delta t$, respectively. The satellite rotated by ~0.23° with every capture; this differs by 0.02° from the theoretical estimation for perfect target-pointing, and $B$ is the baseline, the distance traveled by DIWATA-1 after $\Delta t$. Also shown here is a point $P$, which is a point seen in both Images 1 and 2. (**b**) Shows how the common point $P$ is projected on Image 1, ${P}_{1}$, and Image 2, ${P}_{2}$, where $Q(t)$ and $Q(t+\Delta t)$ are the positions of the optical center of DIWATA-1 at time $t$ and $t+\Delta t$, respectively, and ${e}_{1}$ and ${e}_{2}$ are the epipoles of Images 1 and 2, respectively. The line passing through the projection of point $P$ and the epipole is called an epipolar line.

The DIWATA-1 target-pointing capability was also utilized in this study; this allows the microsatellite to point and lock the cameras to view a specific location as it moves through its orbit. Although stereoscopy-based measurement of cloud-top heights can be performed with a camera in a steady position, as in the case of geosynchronous satellites, DIWATA-1 traverses its orbit at an approximate speed of 7.6 km/s. Thus, it is necessary to use its target-pointing capability so that the same scene and location are apparent in the captured images. DIWATA-1 rotates by an angle Δ*θ* as it traverses its orbit. Thus,

1 $$\Delta \theta =|\theta (t)-\theta (t+\Delta t)|,$$

where Δ*t* is the time difference between two consecutive captures set to 200 ms. Theoretically, the angle *θ* (see Fig. 1a) is subtended by the point directly below the satellite (i.e., the subsatellite point), and the central coordinate of the image is given by the following equation:

2 $$\theta (t)=\arctan \left[\frac{D(t)-I(t)}{Z}\right]\times \frac{180^\circ }{\pi },$$

where $D(t)$ is the along-track position of the subsatellite point at time *t*, $I(t)$ is the central coordinate of the image captured at time $t$, $Z$ is the satellite altitude relative to the subsatellite point, and $\theta (t)$ is the view zenith angle at time $t$. Note that this equation is valid only when the curvature of the Earth is negligible.

### Observations

**Figure 2:**
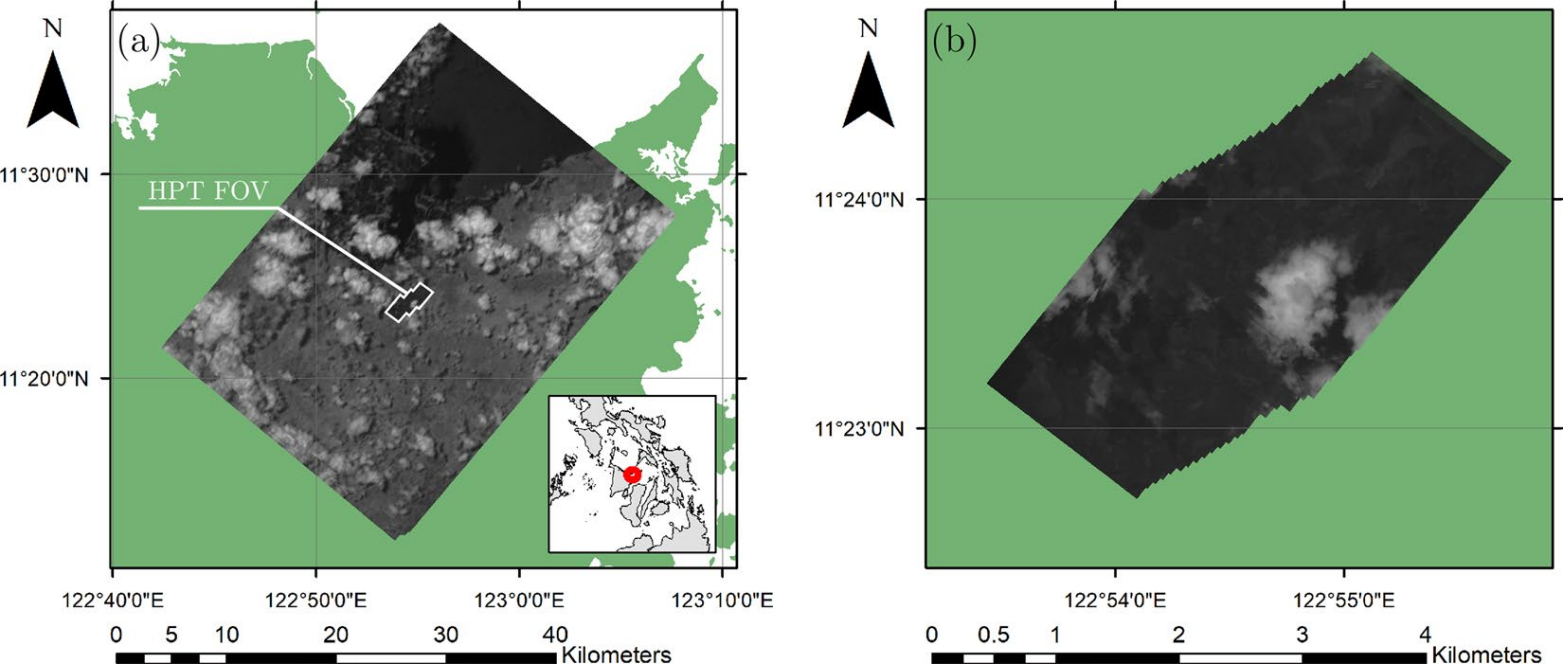
Images captured by (**a**) SMI and (**b**) HPT from 12:40:39 to 12:40:45 PST on 5 August 2017, stacked from left to right. The HPT FOV is overlaid on (a), enclosed by a white line.

On August 5, 2017 DIWATA-1’s SMI and HPT successfully captured clouds hovering above Iloilo province in the Philippines from 12:40:39 to 12:40:45 Philippine Standard Time (PST) or 04:40:39 to 04:40:45 Coordinated Universal Time (UTC). To minimize the cloud advection between each image, the interval between each capture was set to the minimum possible value of 200 ms. In accordance with the total capture time, each camera obtained a total of 30 images. Most clouds captured within the fields of view (FOVs) of the camera seem to have been low-lying, and potentially stratocumulus or cumulus (Fig. 2). Since its release, the satellite’s altitude has decreased to 378.6 km around the time the images were acquired for this study.

The images were then georeferenced, using surface features as control points, using ESRI ArcGIS 10.2.2, and the coordinates of each pixel in the image were estimated. Figure 2 shows the georeferenced images captured by the SMI and HPT via target-pointing. The HPT FOV is overlaid on the image captured by the SMI and is enclosed with a white line. The center of the initial SMI image was located at approximately 11.41°N and 122.91°E. The final image had a central position of 11.42°N and 122.93°E.

To find the rotation of the satellite due to target-pointing (see Eqs. 1 and 2), the two-line element dataset of DIWATA-1 was entered into the orbital calculation software Systems Tool Kit (STK) 11.6 (developed by Analytical Graphics, Inc.) to extract $D$ and $Z$. For perfect target-pointing, that is $I(t)=I(t+\Delta t)$ (see Fig. 1a), the average value of Δ*θ* between successive images is nominally 0.21°. However, as the target-pointing encounters an error at a certain level, that is, $I(t)\ne I(t+\Delta t$), the area captured by the telescope on the satellite shifts slightly from one location to another. Using the central coordinates of the captured images while retaining the values for *D* and *Z*, Δ*θ* is, on average, ~0.23° from one location to another; this is a difference of 0.02° from the theoretical estimate. Hence, the image centers are displaced by approximately 84 m, with the SMI and HPT image centers being displaced by approximately 1.5 and 28 pixels, respectively. This displacement is not a significant problem as there are overlaps exceeding 99% and 94% between consecutive SMI and HPT images, respectively.

On the same day as these images were taken, the Japanese weather satellite HIMAWARI-8 captured the east-southeast Asian region at 12:40:00 PST, 39 seconds before DIWATA-1 captured the clouds above Iloilo. HIMAWARI-8 has 16 spectral bands: 3 in the visible, 3 in the near-infrared, and 6 in the far-infrared region^39^. The spatial resolution at the subsatellite points in the blue, green, and first near-infrared (0.86 μm) bands is 1 km; for the red, it is 0.5 km, and for the other remaining bands the spatial resolution is 2 km. The cloud-top height product on HIMAWARI-8 uses the brightness temperature measured by one of its far infrared bands, Band-14 (11.2-μm)^21^; this study compares that to DIWATA-1. Here, the brightness temperature is converted to cloud-top height using a radiosonde measurement (see details in Appendix C).

## Method

**Figure 3:**
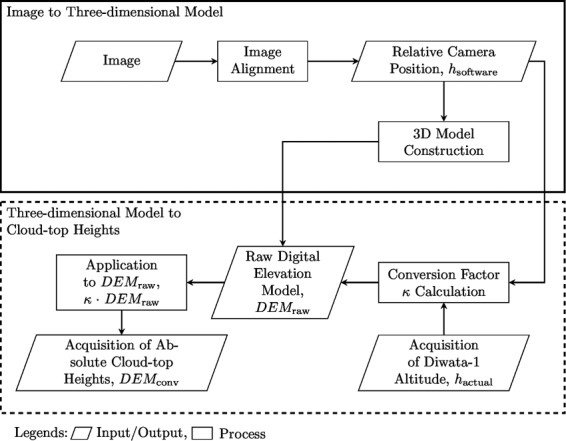
Analysis procedure for cloud-top height estimation from stereoscopic image set. The upper and lower sections show the construction of the three-dimensional model from the images and estimation of the cloud-top heights from the three-dimensional model, respectively.

The three-dimensional model of the scene used to estimate the cloud-top height was generated from a set of stereoscopic images in a Multi-view Stereo (MVS) algorithm-based^40,41^ software package RealityCapture 1.0.3.6310 RC, developed by Capturing Reality; this process is shown in Fig. 3 and is mainly composed of two workflows. First, a three-dimensional model of the scene was constructed from the stereoscopic images captured by DIWATA-1 (upper portion of Fig. 3 workflow, enclosed by the solid rectangle); this was then used to estimate cloud-top heights (bottom portion of Fig. 3 workflow, enclosed by the dashed rectangle).

In the “Image Alignment” block, the images were aligned based on their cloud and terrain surface features, whose relative positions change in each image due to the movement of DIWATA-1. Assuming that the cloud advection and the change in cloud morphology is negligible, the camera movement from one point to another could be estimated from the differences of the feature positions; therefore, the camera position and/or orientation^42^, which is the “Relative Camera Position” output block in Fig. 3, could be determined. The next block, “3D Model Construction”, constructs a three-dimensional model from the images by triangulating three-dimensional points and estimating a potential surface patch from the features that lie within a set number of pixels from the same epipolar line^43^ (see Fig. 1b). The maximum set of pixels was set to one, i.e., features that lie more than one pixel from the same epipolar line are discarded by the software and are not used to create the model. These patches then form a surfel (surface element) model^43^.

**Figure 4:**
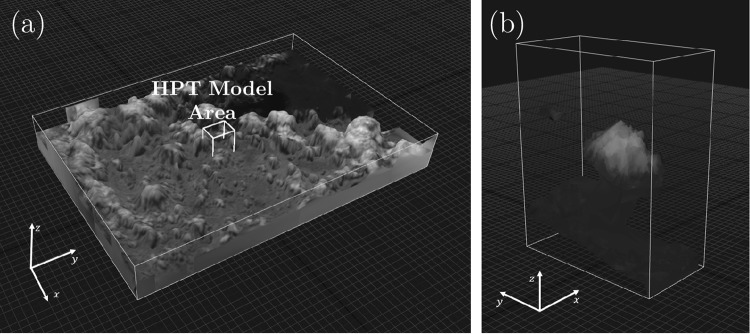
Three-dimensional models constructed from (**a**) SMI and (**b**) HPT images. The approximate HPT FOV is overlaid on (a), enclosed in a white box. The axes on the lower-left indicate the model orientation.

The camera focal length was also set to further assist the software with image alignment. In this process, as the software requires 35-mm equivalent focal lengths ${f}_{35{\rm{mm}}}$ as input, the camera focal lengths were converted according to the following relationship^44^:

3 $${f}_{35{\rm{mm}}}=\frac{43.27{\rm{mm}}}{S}\times f,$$

where 43.27 mm is the diagonal size of a film with 35-mm height, $S$ is the diagonal size of the SMI/HPT sensor, and $f$ is the camera focal length. The diagonal size of the SMI and HPT sensors is 6.09 mm, and their focal lengths are 48.2 and 974 mm, respectively. Using Eq. 3, the 35-mm equivalent focal lengths calculated are 0.342 m and 6.92 m for the SMI and HPT, respectively. Entering these values into the software will calibrate the estimated camera positions of the software. However, this method was not applied to the HPT due to its large ${f}_{35{\rm{mm}}}$, rather only the raw HPT images were used without additional inputs, and follows the workflow shown in Fig. 3. The results of the three-dimensional model of the SMI, with the assistance of ${f}_{35{\rm{mm}}}$, and of the HPT, are shown in Fig. 4a,b, respectively. The time it took for the software to align the images until to giving the model texture is approximately 1.5 minutes for the SMI and 35 seconds for the HPT for a Windows 10 PC with Intel Core i7–6700HQ CPU at 2.6 GHz, a RAM of 8.00 GB, and a graphics card of NVIDIA GTX950M. The software measures height and estimated camera positions in “units” by default; these are measured relative to the internal coordinate system of the software as depicted by the gridded surface in Fig. 4. For the sake of discussion, these units will be called “software units.” The models were made using 30 images for both SMI and HPT, and the estimated camera altitudes of the software range from 420.3 to 420.8 software units and from 4,122.4 to 4,154.3 software units for the SMI and HPT models, respectively. Compared to the raw images from DIWATA-1, the number of pixels along the x- and y-directions increased to 1,113 by 800 pixels and 1,060 by 638 pixels for the SMI and HPT models, respectively. Furthermore, because all DIWATA-1 images do not show what is below the clouds, the three-dimensional models do not include cloud-base height data. Exporting the three-dimensional models from the software results into raw digital elevation maps ($DE{M}_{{\rm{raw}}}$) or surface elevation maps, where each point in the map has a height, with respect from the gridded surface (expressed in software units), the clouds can be identified as relatively high points on the DEM or through visual assessment. These estimated camera positions and the heights of each pixel in the DEM are necessary to convert the measurements from software units into meters or kilometers.

In the “Conversion Factor Calculation” block, a relationship between the estimated camera altitudes from the software, expressed in software units, and the altitude of DIWATA-1, expressed in meters, at the time of acquisition. The conversion factor for the SMI and the HPT is given by the following:

4 $$\kappa =\frac{{h}_{{\rm{actual}}}}{{h}_{{\rm{software}}}},$$

where ${h}_{{\rm{actual}}}$ is the altitude of DIWATA-1 generated by the STK software, expressed in meters, which differs by around 1 km from the actual altitude of the microsatellite, while ${h}_{{\rm{software}}}$ is the altitude of the camera as estimated by RealityCapture, expressed in software units. However, because the camera positions estimated by the software for the HPT were not calibrated using its ${f}_{35{\rm{mm}}}$ focal length, the value of ${h}_{{\rm{software}}}$ for the HPT were instead calibrated using the default 35-mm focal length output of the software, *f* ′_35mm_. The details of this process are presented in Appendix A. These results into a conversion factor $\kappa$ of meters per software unit, which is then multiplied to $DE{M}_{{\rm{raw}}}$ to convert the measurements from software units to meters, i.e., $DE{M}_{{\rm{conv}}}=\kappa \cdot DE{M}_{{\rm{raw}}}$, where $DE{M}_{{\rm{conv}}}$ is the DEM with height measurements taken in meters. The number of conversion factors $\kappa$ in a single DEM will be equal to the number of images used. In this study, the average of 30 conversion factors corresponding to the 30 images were used for both the SMI and HPT.

The resulting cloud-top heights from the stereoscopic technique were compared to those of the cloud-shadow technique and to the clouds’ brightness temperature. The purpose of this comparative analysis is to confirm whether the cloud-top height measurements from the stereoscopic method are consistent with those derived using other techniques. For the cloud-shadow technique, the height of a cloud-edge identified in the $DE{M}_{{\rm{conv}}}$ was compared to the corresponding cloud-edge whose height was estimated based on the shadow cast by the cloud and solar angles. The details of this procedure are presented in Appendix B. On the other hand, the brightness temperature approach uses the brightness temperature measured by HIMAWARI-8’s Band-14 (11.2-μm) and the temperature vertical profile measured by a radiosonde to derive the cloud-top heights. The details of this procedure are presented in Appendix C.

## Results and Discussion

### Cloud-top heights from stereoscopic technique

**Figure 5:**
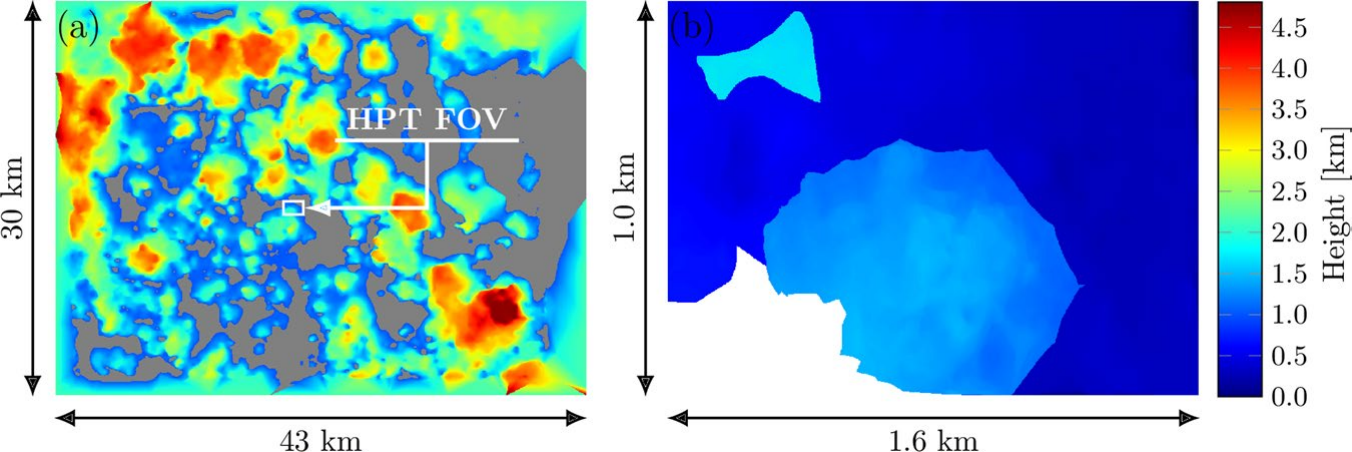
Cloud-top height results for (**a**) SMI and (**b**) HPT, converted from software units to meters. The model sizes differ from the camera FOVs because the models are a combination of multiple images. Areas that are colored gray are cloud-free regions.

The images of clouds captured on August 5, 2017 at 12:40:39 to 12:40:45 PST were aligned to retrieve the estimated camera positions from RealityCapture. With the STK-simulated positions and altitudes of DIWATA-1 at the capture time, the conversion factor $\kappa$ (Eq. 4) was estimated to measure the cloud-top heights in $DE{M}_{{\rm{conv}}}$. The average conversion factor for the SMI is $\kappa =876.1\pm 2.3\,$m per software unit, while the HPT has $\kappa =89.2\pm 0.2$ m per software unit. Figure 5a,b show the digital elevation map $DE{M}_{{\rm{conv}}}\,$for the SMI and HPT, respectively. From Fig. 5a, the estimated cloud-top heights range from 1.5 km to 4.5 km, which is indicative of low clouds and developed cumulus clouds and confirms the visual assessment carried out in Section 2.2. Figure 5b, however, shows a closer inspection of clouds with heights ranging from 1.5 km to 2.0 km, which are also low. The masked region in Fig. 5b, covered in white, contains erratic cloud-top height values due to the lack of overlapping features within this region, which is due to the target-pointing error. The full image of the HPT is shown by Figure S1 in the supplementary material. From $DE{M}_{{\rm{conv}}}$, the vertical resolutions were found to be 40 m and 2 m for the SMI and HPT, respectively.

With the baseline $B$, i.e. the distance traversed by the satellite between the first and last images, set at 45.6 km, just as the focal lengths of the SMI and HPT and altitude of DIWATA-1 were (see Table 1), the theoretical error $\Delta h\,$in the constructed DEM is as follows^18,45^:

5 $$\Delta h=\frac{{H}^{2}}{fB}\times \Delta p$$

where $H$ is the altitude of DIWATA-1, $f$ is the focal length, and $\Delta p$ is set to $7.4\,{\rm{\mu }}{\rm{m}}$, which is the estimated disparity error for both SMI and HPT cameras. The resulting $\Delta h$ ranges from 0.48 km to 0.49 km, and 0.024 km for the SMI and HPT DEMs, respectively, accounting the simulation error of DIWATA-1’s altitude and if the cloud advection and change in morphology is negligible. Otherwise, the disparity error may need to be adjusted, which would result in errors of higher multiples of 0.48 km and 0.024 km or a complete failure of image alignment. Another possible source of error for the $DE{M}_{{\rm{conv}}}$ is the imperfect target-pointing (see Section 2.2), which is off by 0.02° from the theoretical estimate. Since DIWATA-1 was approximately 20 km away from the center of the target at the final image capture, this could translate to an error of 1.1 km in the cloud-top height measurements^46^.

### Comparison of the Stereoscopic-based Cloud-top Heights with that from other Techniques

**Figure 6:**
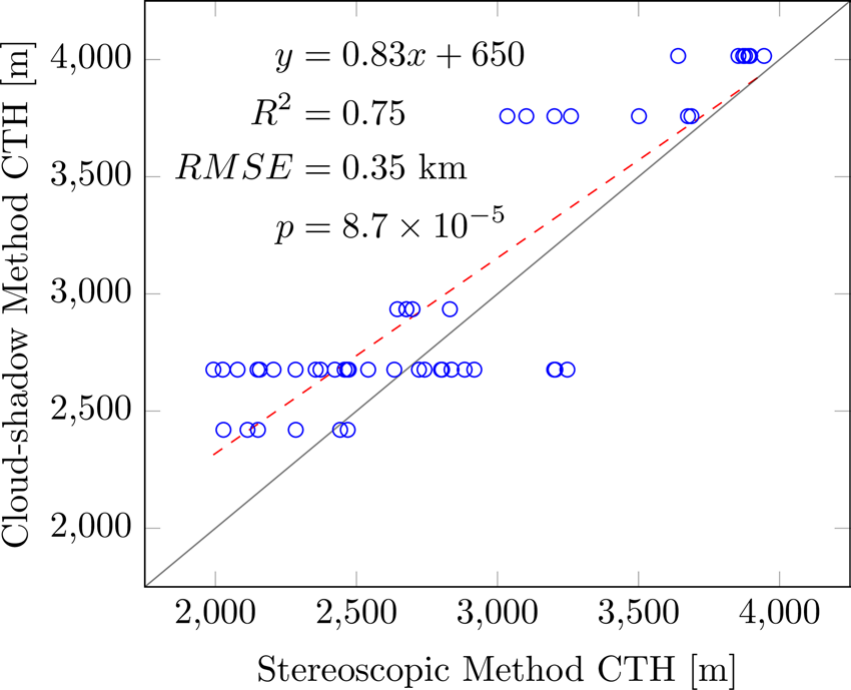
Correlation between the Cloud-top Height results of Cloud-shadow and Stereoscopic Techniques in the SMI. The solid gray line is the $y=x$ line, which was overlaid for reference.

The cloud-top heights derived from the stereoscopic technique were compared to the cloud-shadow-derived cloud-top heights. Measuring the cloud-edge heights through cloud-shadow technique requires the solar angles (Appendix B). The solar zenith and azimuth angles throughout the duration of DIWATA-1’s operation are, on average, $12.5^\circ$ and $297.5^\circ$, respectively. Upon georeferencing, the images were rotated by ${\epsilon }=309^\circ$ or $-51^\circ$ from their original orientation. From Eq. 11, the solar azimuth angle with respect to the original orientation of the images *β* is approximately $348.5^\circ$ or $-11.5^\circ$. This value was used to pair cloud-edge pixels to their corresponding shadow. Figure 6 shows a scatter plot of the estimated cloud-edge heights from the cloud-shadow technique and the cloud-edge heights estimated from $DE{M}_{{\rm{conv}}}$ for the SMI. Multiple cloud-edge points were gathered and correlated for the SMI. The cloud-shadow measurements correlate strongly with that of $DE{M}_{{\rm{conv}}}$, with an ${R}^{2}=0.75$ and a significance value $p=8.7\times {10}^{-5}$, with a root-mean-square error (RMSE) value of 0.35 km; most of the outliers are from thin and relatively small clouds. On the other hand, the average difference between the stereoscopy-derived and the cloud-shadow-derived cloud-top heights for the HPT is ~30 m.

The cloud-top height from the $DE{M}_{{\rm{conv}}}$ of SMI was also compared to HIMAWARI-8 Band-14 (11.2-μm) brightness temperature data captured at 12:40:00 PST, 39 s before DIWATA-1 captured the same scene. Cloud-top height estimation from the HIMAWARI-8 data was performed using brightness temperature conversion (see details in Appendix C). The resulting cloud-top height from this technique is consistent with the HIMAWARI-8 cloud-top height standard product distributed by JAXA Himawari Monitor. The spatial resolution of the standard product is ~5.0 km, which is ~100 times larger than the spatial resolution of SMI and 2.5 times larger than HIMAWARI-8 Band-14, making most of the clouds seen by SMI unresolvable. By using a radiosonde measurement, the cloud-top height can be derived at a higher spatial resolution.

**Figure 7:**
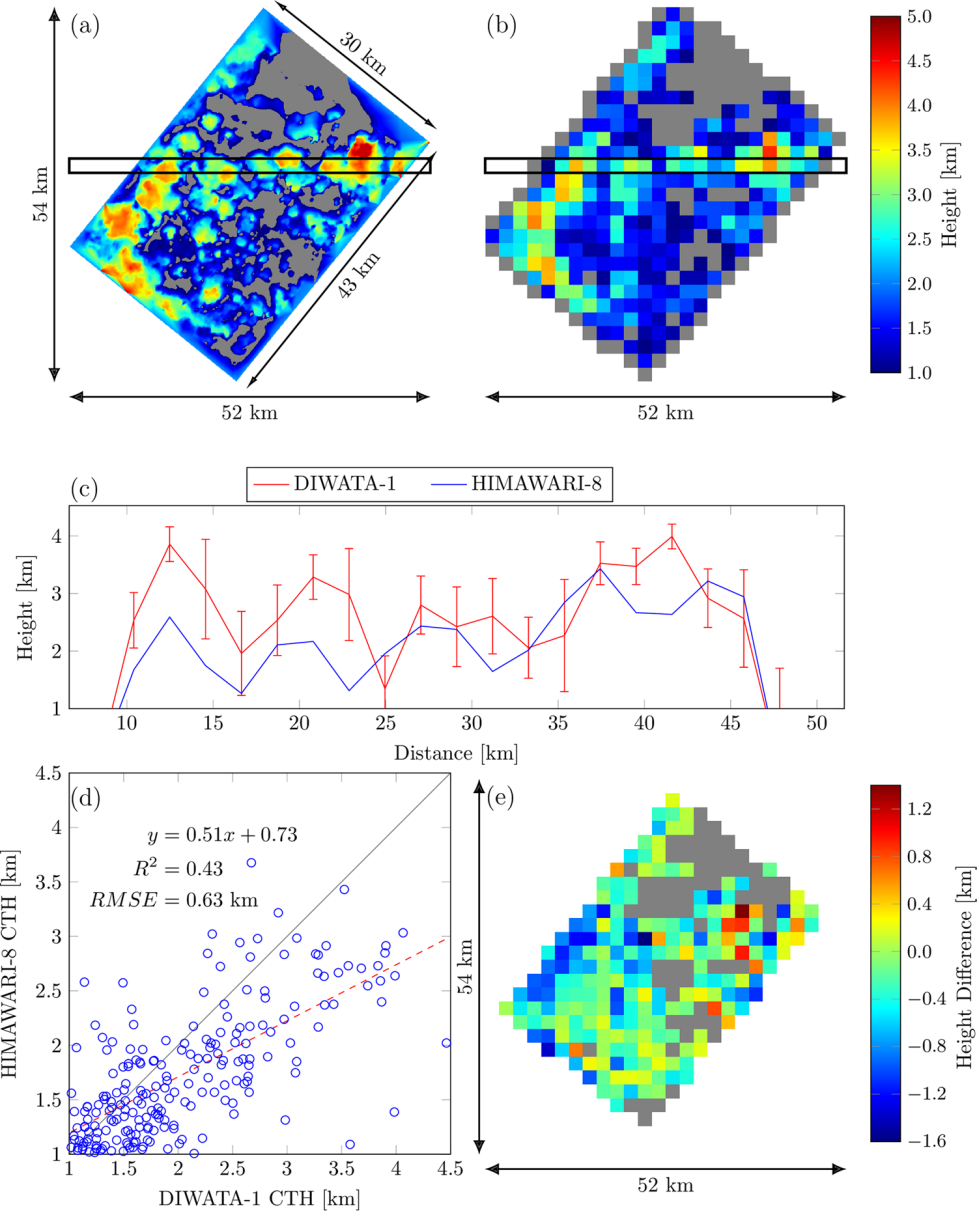
Comparison of (**a**) DIWATA-1 SMI and (**b**) HIMAWARI-8 cloud-top height results, where the gray areas are relatively cloud-free pixels. (**c**) Cross-section of cloud-top heights in (a) and (b) for region between black horizontal lines (Himawari 8/9 gridded data are distributed by the Center for Environmental Remote Sensing (CEReS), Chiba University, Japan); (**d**) correlation between HIMAWARI-8 and SMI, where the solid gray line is the $y=x$ line, which was overlaid for reference; and (e) Cloud-top height difference between the upscaled SMI and HIMAWARI-8.

Atmospheric vertical profiles observed by radiosondes flown over Cebu, 150 to 200 km from Iloilo, were used in conjunction with the HIMAWARI-8 data. High-resolution radiosonde data, averaged over the period August 11–20, 2013, were employed^47^ to represent atmospheric conditions during the time that DIWATA-1 was acquiring its images. Figure 7 shows a comparison of the cloud-top heights obtained from the DIWATA-1 SMI (Fig. 7a), which was rotated to match the orientation of the HIMAWARI-8 data (Fig. 7b), as well as a cross-sectional comparison of the two (Fig. 7c). The section being compared is bounded by the two horizontal black lines in Fig. 7a,b. This cross-sectional region was chosen because majority of the pixels within this area are cloudy and have non-zero cloud-top heights. The DIWATA-1 cloud-top height measurements shown in Fig. 7c were obtained by averaging values within a region of 49 × 49 pixels of the rotated SMI model (Fig. 7a), which corresponds to a single pixel of the HIMAWARI-8 data.

It is possible that the vertical temperature profile in Cebu may have differed from that at the SMI imaging location. However, data from the Atmospheric Infrared Sounders (AIRS) satellite^48^ indicates that there is little difference between the vertical profiles of Cebu and Iloilo; the maximum difference is ~0.8 K, which translates to a height difference of less than 50 m.

Figure 7c clearly shows that the cloud-top heights estimated from the DIWATA-1 SMI $DE{M}_{{\rm{conv}}}$ are greater than that from the HIMAWARI-8 data, which is further verified by Fig. 7d,e. Figure 7d shows the correlation between the cloud-top heights measured by HIMAWARI-8 and SMI, and Fig. 7e shows the differences in HIMAWARI-8 and the upscaled DIWATA-1 cloud-top height measurements, where a negative difference indicates a higher DIWATA-1 cloud-top height. The negative differences in the middle are due to the unresolved clouds from HIMAWARI-8. The slope of the regression line in Fig. 7d between the two measurements is less than one, which further indicates that DIWATA-1 measurements are generally higher than those from HIMAWARI-8, with both measurements having an ${R}^{2}$ of 0.43 and an RMSE of 0.63 km. Overall, the absolute difference in cloud-top heights has an average of 0.15 km with a standard deviation of 0.30 km. One of the possible sources of this difference is the conversion of the HIMAWARI-8 brightness temperature to cloud-top heights, which was only achieved by converting the former to the latter directly using an atmospheric vertical profile. This method can be improved by further analyzing the consistency between the brightness temperature measured by satellites and that of radiosondes, which could be a subject of future studies. However, it has been reported that the cloud-top height product of HIMAWARI-8 can be under-estimated compared to the results from the Moderate Resolution Imaging Spectroradiometer (MODIS) and the Cloud-Aerosol LIDAR and Infrared Pathfinder Satellite Observations (CALIPSO)^49^. Another possible explanation is the low spatial resolution of the HIMAWARI-8 Band-14, which is ~2.0 km, compared with that of the DIWATA-1 SMI, which is 58.1 m^7^.

## Conclusions

Using data from DIWATA-1, this study constructed three-dimensional cloud models (DEMs) at higher spatial and temporal resolutions than those previously reported^7,9,11,13,15–17,21,22,25,26^. Cloud-top heights were measurable for 6-second observations with a 200-ms interval between captures, with 40-m and 2-m vertical resolutions for SMI and HPT, respectively. The estimated RMSE with the cloud-edge height determined using the cloud-shadow technique for the SMI is 0.35 km and has a correlation coefficient of 0.75 when compared with DIWATA-1. The maximum difference of 30 m between the cloud-shadow derived and stereoscopy-derived cloud-top heights on the HPT might have been due to the lack of variation in the sample points from the cloud-shadow cloud-edge height measurements. An average difference of 0.15 km, with a maximum difference of 1.7 km relative to a rough estimation from the HIMAWARI-8 Band-14 data was also obtained. Comparison with other cloud-top measuring satellites such as MODIS, CALIPSO, MISR, LIDARs, and others that can measure cloud-top heights can be done to further assess the technique presented in this study.

This study developed a novel and cost-effective approach to direct cloud-top height estimation using a microsatellite. By optimizing the minimum images required to construct a DEM, several subsets of images may be extracted from a single set captured from a single pass of the microsatellite. These subsets may then construct multiple DEMs and reflect the cloud-top height from the time interval between each subset; this will then measure the vertical development of the clouds. However, the baseline will become shorter for each DEM, increasing the possible error in the cloud-top height measurement; therefore, it is desirable to have a constellation of microsatellites with capabilities similar to DIWATA-1 as this will decrease the error due to the short baseline. This has been the practice to date, with two or more geostationary satellites for measuring cloud-top heights stereoscopically. The ability to obtain high spatial and temporal resolutions of cloud-top height is crucial for monitoring the development of thunderstorms and understanding severe weather phenomena^50^.

### Appendix A: Calibration of Camera Altitudes for the HPT

First, the optical system of the HPT was assumed to obey the thin-lens approximation, where the distance between the object and the lens is interpreted as the altitude of DIWATA-1. To utilize the altitude of DIWATA-1 as a basis for converting software units to absolute units, like that of the SMI model, the distance between the cameras and the internal coordinate system of the software should be known. However, as the focal length outputs of the software are expressed in 35-mm equivalent focal lengths, the indication is that the distance between the camera and the internal coordinate system of the software is based on ${f}_{35{\rm{mm}}}$. To resolve this, the focal length outputs of the software $f{\text{'}}_{35{\rm{mm}}}$ were converted to actual focal lengths using the inverse of Eq. 3,

6 $$f\text{'}=\frac{f{\text{'}}_{35{\rm{mm}}}}{43.27\,{\rm{mm}}}\times S,$$

where $f\text{'}$ is the equivalent focal lengths of ${f}_{35{\rm{mm}}}^{\text{'}}$. By knowing the relationship between the focal length of the HPT and $f\text{'}$ the distance between the cameras and the model in the software based on $f\text{'}$ can be determined. The relationship between ${f}_{{\rm{HPT}}}$ and $f\text{'}$ is defined to be

7 $$\begin{array}{c}n=\frac{f\text{'}}{{f}_{{\rm{HPT}}}},\\ =\frac{f\text{'}}{974\,{\rm{mm}}}\end{array}$$

Therefore,

8 $$\begin{array}{c}\frac{1}{{f}_{{\rm{H}}{\rm{P}}{\rm{T}}}}=\frac{1}{{d}_{o,{\rm{H}}{\rm{P}}{\rm{T}}}}+\frac{1}{{d}_{i,{\rm{H}}{\rm{P}}{\rm{T}}}},\\ \frac{1}{n\cdot {f}_{{\rm{H}}{\rm{P}}{\rm{T}}}}=\frac{1}{n\cdot {d}_{o,{\rm{H}}{\rm{P}}{\rm{T}}}}+\frac{1}{n\cdot {d}_{i,{\rm{H}}{\rm{P}}{\rm{T}}}},\\ \frac{1}{{f}^{{\prime} }}=\frac{1}{{d}_{o}}+\frac{1}{{d}_{i}}\end{array}$$

where ${d}_{o,{\rm{HPT}}}$ is the altitude of DIWATA-1, ${d}_{i,{\rm{HPT}}}$ is the distance between the HPT lens and image sensor, while ${d}_{o}$ and ${d}_{i}$ are the distances of the object and image, respectively, in relation to $f\text{'}$. By multiplying $n$ to the altitude of DIWATA-1, the altitude of the cameras relative to $f\text{'}$ can be determined. The input for ${h}_{{\rm{software}}}$ for Eq. 4 will then be $\frac{n}{{h}_{{\rm{software}}}}$, or simply ${h}_{{\rm{actual}}}$ is ${d}_{o}$.

### Appendix B: Cloud Identification and Height Estimation using the Cloud-Shadow technique

The values of the cloud-top heights from $DE{M}_{{\rm{conv}}}$ were compared to a cloud-top height derived using the cloud-shadow technique, which requires the solar zenith, azimuth angles and distance between the cloud-edge and the edge of its shadow.

**Figure 8:**
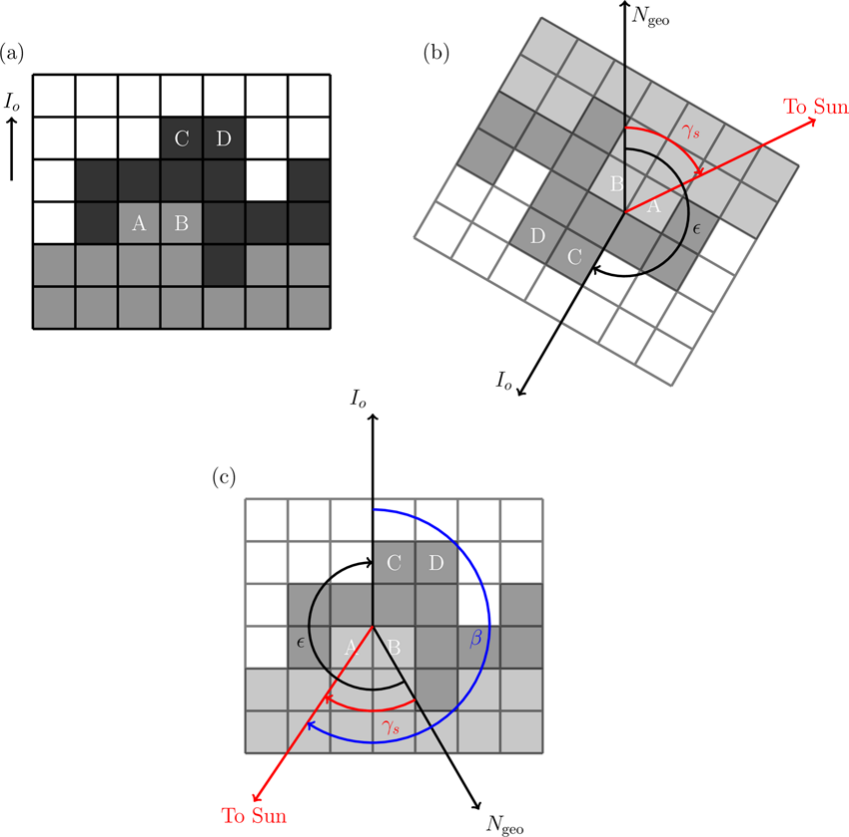
(**a**) Sample set of image pixels with the gray-filled squares as clouds and black-filled squares as their corresponding shadow; (**b**) Orientation of the image after georeferencing where *γ*_*s*_ is the solar azimuth angle relative to the geographic north and ε as the angle between the geographic north and the original orientation of the image; and (**c**) is the original orientation of the image overlaid with the direction of the geographic north and solar azimuth angle from the geographic north. The angle *β* is the solar azimuth angle relative to the original orientation of the image.

Figure 8a illustrates a cloud and shadow image (pixels are shown as boxes in a grid), where gray-filled boxes are cloud pixels and black-filled boxes are shadow pixels corresponding to the cloud pixels. The first stage to properly match a cloud edge to its shadow, i.e., to know whether the pixel labeled “A” matches with pixel “C” or “D”, uses the solar azimuth angle.

The solar zenith ${\alpha }_{s}$ and azimuth angles ${\gamma }_{s}$ were estimated by the following^51,52^:

9 $$\sin ({\alpha }_{s})=\,\cos (\delta )\cos (\phi )cos(\omega )+\,\sin (\delta )\sin (\phi )$$

10 $$\cos ({\gamma }_{s})=\frac{\sin (\delta )\cos (\phi )-\,\cos (\delta )\sin (\phi )\,\cos (\omega )}{\cos ({\alpha }_{s})}$$

where $\delta$ is the solar declination, $\phi$ is the latitude coordinate of the target, and $\omega$ is the hour angle, where all three parameters are expressed in degrees.

The direction of the cloud shadow should be 180° away from the solar azimuth angle, which is relative to the geographic north. To know the orientation of the geographic north ${N}_{{\rm{geo}}}$ relative to the original orientation of the image ${I}_{{\rm{o}}}$, the image should first be georeferenced (Fig. 8b). Where the angle between ${N}_{{\rm{geo}}}$ and ${I}_{{\rm{o}}}$ (the angle how much the image has rotated) is indicated by ε (Fig. 8c) given the convention that clockwise or north-to-east rotation is positive. The solar azimuth angle relative to ${I}_{{\rm{o}}}$, $\beta$, can be estimated as follows:

11 $$\beta =360^\circ -{\epsilon }+{\gamma }_{s}.$$

Therefore,

12 $$|\tan (\beta )|=\frac{|{A}_{x}-{C}_{x}|}{|{A}_{y}-{C}_{y}|}$$

where ${A}_{x}\,$and ${C}_{x}$ are the x-coordinates of pixel “A” and “C”, respectively, and ${A}_{y}$ and ${C}_{y}$ are the y-coordinates of the same pixels, respectively. At times when the shadow pixel is difficult to identify due to the spatial resolution of the camera, the horizontal and vertical pixel distance combination with the value nearest to $\tan (\beta )$ will be chosen. In cases where $\beta =90^\circ$ or $270^\circ$, the shadow pixel is directly left or right of the cloud pixel, respectively, and if $\beta =0^\circ$ or $180^\circ$, the shadow pixel is directly below or above the cloud pixel, respectively.

Using $\beta$ and the original orientation of the image to count the horizontal and vertical pixel distances between a cloud and a shadow removes the possible changes in the ground sampling distance (GSD) of the image caused by the rotation of the georeferencing. However, because there are cases in which the cloud and/or the cloud shadow pixel contaminates their surrounding pixels, it is possible that the cloud or cloud shadow is actually located at the neighbors of the chosen pixel. To account for this uncertainty, the height error of a cloud-edge estimated from this technique corresponds to a single pixel.

After identifying the shadow pixel, the distance ${d}_{{\rm{cs}}}$ between the cloud and shadow pixel is estimated by the Euclidean distance multiplied by the GSD of the image as below,

13 $${d}_{{\rm{cs}}}={\rm{GSD}}\times \sqrt{{({A}_{x}-{C}_{x})}^{2}+{({A}_{y}-{C}_{y})}^{2}}$$

The height of the cloud edge pixel ${h}_{{\rm{c}}}$ is then estimated via,

14 $${h}_{{\rm{c}}}={d}_{{\rm{cs}}}\,\tan (90^\circ -{\alpha }_{s}).$$

### Appendix C: Conversion of the Brightness Temperature of HIMAWARI-8 to Cloud-top Heights

The cloud-top heights measured using the DIWATA-1 stereoscopic technique was compared with those obtained using HIMAWARI-8. To properly compare the two, the brightness temperature measured by HIMAWARI-8 was converted to cloud-top height using a radiosonde sounding.

The radiosonde used is the Vaisala RS41, which measures the temperature profile of the atmosphere as a function of altitude (the uncertainty of the instrument for measuring altitude is 10 m while for temperature it is 0.3 °C^53^), $\,T(z)$ where $z$ is the altitude of the radiosonde, expressed in meters from sea level, and $T$ is the temperature expressed in degrees Celsius at altitude $z$. The temperature profile was measured every 2 s in the raw data and converted to a range from 25 m to 16,500 m—the temperature of the atmosphere at this range is inversely proportional with altitude—with a step size of 25 m.

Given a brightness temperature $BT$ measured by HIMAWARI-8’s Band-14 (11.2-μm), there is an interval

$$T({z}_{i})\ge BT > T({z}_{i+1}),\,{\rm{where}}\,{z}_{i} < {z}_{i+1}$$

where $i$ is the i-th data point in the radiosonde data. Under the assumption that the change in temperature between ${z}_{i}$ and ${z}_{i+1}$ is linear, the corresponding altitude of $BT$, ${z}_{BT}$, can be found through linear interpolation.

$${z}_{BT}=\left[\frac{{z}_{i+1}-{z}_{i}}{T({z}_{i+1})-T({z}_{i})}\right][BT-T({z}_{i})]$$

However, cloud-top height errors arising from this technique lie in the similarity between the radiosonde-measured cloud-top temperature and satellite-measured cloud-top brightness temperature. Differences can range from less than 2.5 °C to 30 °C for warm clouds^54^, which is the case in this study, and cloud-top height measurements can differ typically by 1.5 km^55^.

To collocate $DE{M}_{{\rm{conv}}}$ from SMI with HIMAWARI-8’s Band-14 (11.2-μm), it was upscaled to match the 2-km spatial resolution of HIMAWARI-8 after orienting it at the same orientation as the HIMAWARI-8 data.

## Supplementary information

Supplementary Information.
