# Supplementary material for: Determination of Cloud-top Height through Three-dimensional Cloud Reconstruction using DIWATA-1 Data
Source: Sci Rep. 2020 May 5;10:7570. doi: 10.1038/s41598-020-64274-z (PMC7200711; doi:10.1038/s41598-020-64274-z)
Supplement: Supplementary file 1 — Supplementary Information. [file 41598_2020_64274_MOESM1_ESM.doc]

**Supplementary Information**

**Determination of Cloud-top Height through Three-dimensional Cloud Reconstruction using DIWATA-1 Data**

Ellison Castro^a,b,*^, Tetsuro Ishida^a,+^, Yukihiro Takahashi^a^, Hisayuki Kubota^a^, Gay Jane Perez^b^, and Joel S. Marciano, Jr.^c,d^

^a^Faculty of Science, Hokkaido University, Sapporo, Japan

^b^Institute of Environmental Science and Meteorology, University of the Philippines Diliman, Quezon City, Philippines

^c^Advanced Science and Technology Institute, Department of Science and Technology, Quezon City, Philippines

^d^Electrical and Electronics Engineering Institute, University of the Philippines Diliman, Quezon City, Philippines

**Correspondence to*: E. C. Castro, Institute of Environmental Science and Meteorology, University of the Philippines Diliman, Philippines.

[*eccastro@stamina4space.upd.edu.ph*](mailto:eccastro@stamina4space.upd.edu.ph)

^+^T. Ishida, Faculty of Science, Hokkaido University, Japan.

*Ishida.tetsuro@sci.hokudai.ac.jp*

**Supplementary**

**Figure S1**


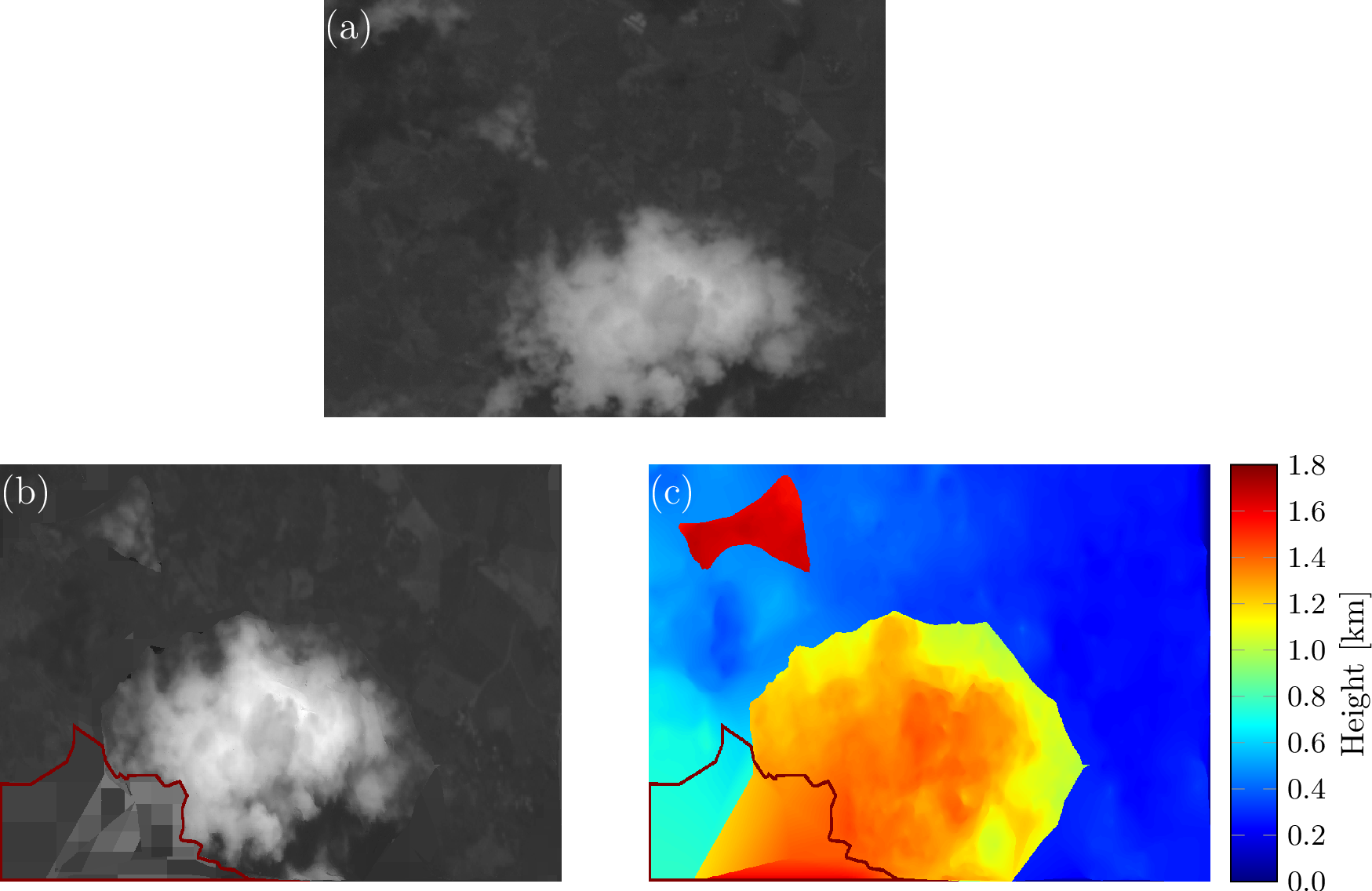


**­­Figure S1:** (a) Raw image captured by the HPT; (b) is rendered textured HPT model. The region enclosed by the red shape was not properly textured by the software, whose pixelated texture is not present in (a), indicating that there are erroneous height values within this region; and (c) shows the digital elevation map (DEM) of the HPT, including the values that were masked in Figure 5.
